# *Laminaria japonica* polysaccharide mitigates acute neuroinflammation in cerebral ischemia-reperfusion injury through Csf3-modulated pathways

**DOI:** 10.3389/fimmu.2026.1801746

**Published:** 2026-04-23

**Authors:** Tangming Guan, Siyu Chen, Dongbo Jiang, Weiming Cai, Huayan Chen, Yan Wang, Kefeng Wu, Xin Zhou

**Affiliations:** 1 Department of Pharmacy, Affiliated Hospital of Guangdong Medical University, Zhanjiang, China; 2 Laboratory of Clinical Pharmacy, Affiliated Hospital of Guangdong Medical University, Zhanjiang, Guangdong, China; 3 Marine Biomedical Research Institution, Guangdong Medical University, Zhanjiang, China; 4 School of Chemistry and Environmental Science, Guangdong Ocean University, Zhanjiang, China

**Keywords:** cerebral ischemia/reperfusion (I/R) injury, CSF3, Laminaria japonica polysaccharide (LJP), neuroinflammation, neuroprotection

## Abstract

**Introduction:**

Microglial hyperactivation-driven neuroinflammation is a major driver of secondary brain injury following cerebral ischemia/reperfusion (I/R), yet effective therapies targeting this pathological process remain limited. Marine-derived sulfated polysaccharides have emerged as promising anti-inflammatory agents, but the therapeutic potential and mechanisms of Laminaria japonica polysaccharide (LJP) in cerebral I/R injury are poorly understood.

**Methods:**

LJP was isolated from Laminaria japonica and structurally characterized via high-performance gel permeation chromatography (HPGPC), Fourier-transform infrared (FT-IR) spectroscopy, and ion chromatography. Its neuroprotective effects were evaluated in a mouse model of transient middle cerebral artery occlusion (tMCAO), with assessments of infarct volume, neurological function, and neuroinflammatory markers. Cross-species transcriptomic analysis identified potential targets, which were validated through *in vivo* and *in vitro* functional assays (rescue experiments with recombinant Csf3 and neutralization assays with Csf3-specific antibodies).

**Results:**

Structural characterization confirmed LJP is enriched in fucose, galacturonic acid, glucuronic acid, and sulfate groups. In tMCAO mice, LJP administration reduced infarct volume, improved neurological function, and suppressed microglial pro-inflammatory polarization, accompanied by decreased levels of interleukin-1b (IL-1b), tumor necrosis factor-α (TNFα), and interleukin-6 (IL-6). Transcriptomic analysis identified granulocyte colony-stimulating factor (Csf3/G-CSF) as a potential LJP-regulated mediator. Recombinant Csf3 partially reversed LJP’s protective effects, while Csf3-specific antibodies failed to enhance LJP’s anti-inflammatory benefits, suggesting Csf3 as an important contributory mechanism.

**Discussion:**

These findings demonstrate that LJP mitigates acute neuroinflammation post-I/R at least partly through Csf3 suppression, highlighting its promise as a novel phytotherapeutic agent for ischemic stroke.

## 1 Introduction

Ischemic stroke is a leading cause of death and long-term disability worldwide, initiates pathological neuroinflammatory cascades that worsen secondary brain injury (1, 2). Central to this process are microglia—the CNS-resident immune cells—which rapidly adopt a pro-inflammatory phenotype post-ischemia, releasing cytotoxic cytokines (e.g., IL-1b, TNFα, IL-6) that drive neuronal apoptosis and hinder functional recovery (3–6). Although thrombolytic agents restore cerebral blood flow, their narrow therapeutic window and inability to address neuroinflammation underscore the urgent need for complementary therapies targeting post-reperfusion injury.

Marine-derived sulfated polysaccharides, particularly fucoidans from brown algae, have gained attention for their immunomodulatory precision and low systemic toxicity (7, 8). Laminaria japonica, a nutrient-rich seaweed traditionally consumed in East Asia, produces a fucoidan-like polysaccharide (LJP) with well-documented antioxidant, antitumor, and immunomodulatory properties (9–11). For instance, Fang et al. demonstrated that LJP modulates macrophage polarization to attenuate inflammatory responses, supporting its potential as an anti-inflammatory agent (12). Despite these attributes, LJP’s therapeutic potential in cerebral ischemia/reperfusion (I/R) injury remains unexplored, particularly its capacity to regulate microglial inflammatory signaling—a key driver of secondary brain damage.

Emerging clinical evidence implicates granulocyte colony-stimulating factor (Csf3/G-CSF)—a cytokine classically associated with neutrophil production—as a dual-edged mediator in stroke pathology (13–17). While essential for hematopoietic recovery, accumulating evidence indicates that Csf3 overexpression in the acute or subacute phase post-stroke correlates with exacerbated neuroinflammation and poor patient outcomes. This detrimental effect is likely mediated through promoting microglial activation, neutrophil adhesion, and diapedesis (17, 18). In contrast, studies suggesting neuroprotective effects of Csf3 primarily focus on chronic phases, highlighting the context-dependent role of this cytokine in stroke pathology. Notably, no marine polysaccharides, including conventional fucoidans, have been linked to specific Csf3 modulation in acute ischemic stroke.

Here, we hypothesized that LJP alleviates cerebral I/R injury by modulating Csf3-mediated neuroinflammatory pathways. Sulfated polysaccharides are well-documented to exert anti-inflammatory effects through interactions with cytokine signaling networks, and Csf3 is known to be robustly upregulated during the acute phase of stroke—findings that led us to reason Csf3 may serve as a potential molecular target of LJP. To test this, we employed a cross-species multi-omics approach, integrating transcriptomic profiling of murine I/R brains with human stroke datasets, which identified Csf3 as an LJP-regulated candidate linked to neuroprotective processes. Through comprehensive *in vivo* and *in vitro* functional validations, we show that LJP suppresses Csf3 expression, which in turn may disrupt microglial M1 polarization, attenuate oxidative stress, and preserve neuronal viability. These findings delineate a unique regulatory axis between LJP and Csf3 signaling in the context of ischemic stroke, offering a distinct mechanistic perspective that complements existing investigations on algal fucoidans, and supports the potential of LJP as a targeted marine-derived candidate for ischemic stroke intervention.

## 2 Materials and methods

### 2.1 Extraction and structural characterization of LJP

Extraction: Fresh *Laminaria japonica* (Zhanjiang, China) was thoroughly washed with running water, dried in an oven at 45 °C to constant weight, and ground into powder using an ultra-micro grinder (40-mesh sieve). The sieved powder was refluxed with petroleum ether (1,200 mL, solid-liquid ratio 1:30) at 50 °C for 2 h. After cooling, the supernatant was discarded, and the residue was dried at 50 °C to obtain defatted powder.

The defatted powder was mixed with distilled water (1,600 mL, solid-liquid ratio 1:40) and extracted at 85 °C for 3 h. The mixture was filtered through gauze, and the extraction was repeated three times. The combined filtrate was concentrated using a rotary evaporator. The crude polysaccharide solution was decolorized with activated carbon (1% w/v, 30 min) and deproteinized using the Sevag method (chloroform: n-butanol = 4:1, v/v). After centrifugation (6,000 rpm, 10 min), the supernatant was concentrated under reduced pressure. Ethanol was added to the concentrate to achieve 20% (v/v) ethanol concentration, followed by overnight precipitation at 4 °C. The precipitate was collected by centrifugation (6,000 rpm, 10 min), resuspended in 80% ethanol, and reprecipitated at 4 °C. The final precipitate was dissolved in distilled water and lyophilized to obtain purified LJP.

The phenol-sulfuric acid method was employed for the quantification of total sugar content in LJP. For the determination of other key components, the Bradford assay was used to measure protein levels, while the barium chloride-gelatin turbidity method was utilized for sulfate group quantification, and the carbazole-sulfuric acid method was applied to assess uronic acid content. To evaluate LJP purity, ultraviolet (UV) spectrophotometry (UV 2450, Shimadzu, Kyoto, Japan) was performed over the wavelength range of 200–400 nm, with specific attention to absorption peaks at 260 nm (nucleic acid marker) and 280 nm (protein marker) to rule out significant contamination by these impurities. Molecular weight distribution was determined via high-performance gel permeation chromatography (HPGPC; LC-10A, Shimadzu Ltd., Japan) equipped with a BRT105-103–101 tandem gel column. Functional group characterization was performed using Fourier-transform infrared (FT-IR) spectroscopy (Nicolet iS 5 FT-IR, Shanghai Lairui Scientific Instrument Co., Ltd.) over the spectral range of 4000–400 cm^-1^. For monosaccharide composition analysis, ion chromatography (ICS-5000, Thermo Fisher Scientific) was employed with a Dionex Carbopac™ PA20 column, using 15 mM sodium hydroxide (NaOH) as the mobile phase for elution.

### 2.2 Animal experiments

Male C57BL/6 mice (9–10 weeks old, 23–27 g) were acquired from Liaoning Changsheng Biotechnology Co., Ltd. They were housed in a controlled environment maintained at 22 ± 1 °C with 55 ± 10% relative humidity, under a 12 - hour light/dark cycle. All animal procedures complied with the National Institutes of Health Guide for the Care and Use of Laboratory Animals and were approved by the Animal Ethics Committee of Guangdong Medical University.

Transient Middle Cerebral Artery Occlusion (tMCAO) Model: Focal cerebral ischemia was induced via a 45 - minute transient middle cerebral artery occlusion (tMCAO), following established protocols (19, 20). To minimize variability from sex - related differences and optimize group sizes, only male mice were used. Mice were randomized into groups using a computer - generated random number sequence, with randomization and coding handled by an independent researcher not involved in data analysis—ensuring blinded experimentation. Surgeries and outcome assessments were performed by personnel unaware of group assignments.

Mice were excluded from endpoint analyses for reasons including death before the endpoint, poor health/drop - out scores (e.g., weight loss, abnormal behavior). Exclusion/inclusion details are in **Supplementary Table 1**. For tMCAO induction, mice were anesthetized with 4% isoflurane (induction) and 2% isoflurane (maintenance), with core temperature maintained at ~37 °C via a servo - controlled heating blanket. A silicon-coated monofilament (Doccol, 602256PK10, Sharon, MA, USA) was inserted to the left common carotid artery to occlude the middle cerebral artery origin for 45 minutes, followed by reperfusion through filament withdrawal. Stroke volumes were assessed 3 days post - tMCAO using TTC staining. Sample sizes were calculated based on preliminary data and literature (19, 21), with post - hoc power analysis (G*Power 3.1) confirming cohort sizes (n = 4–5 per group) provided >90% power to detect primary outcome differences (infarct volume, mNSS).

Pharmacological Interventions: LJP (purified Laminaria japonica polysaccharide, prepared as described in “Extraction and Structural Characterization of LJP”) was dissolved in sterile saline and administered intragastrically once daily for 14 consecutive days prior to tMCAO surgery, with treatment continued postoperatively. Three LJP doses were selected based on preliminary dose-finding data and prior reports (22) investigating the bioactivity of related marine sulfated polysaccharides in rodent models of cerebral ischemia: 100 mg/kg (LJP-L), 200 mg/kg (LJP-M), and 400 mg/kg (LJP-H). Animals in the sham and vehicle groups were administered equivalent volumes of sterile saline following the same dosing schedule. For Csf3 rescue experiments, LJP-treated mice received concurrent subcutaneous injections of recombinant murine Csf3 (250 μg/kg daily, PeproTech, 250-05) starting post-tMCAO. Three consecutive treatments were carried out at 2, 24 and 48 h after surgeries.

Behavioral Tests: Behavioral tests were performed before tMCAO and 72 h after tMCAO. The data of dead mice during the experiment were excluded.

Modified Neurological Severity Score (mNSS): Neurological function was evaluated using the modified Neurological Severity Score (mNSS), a composite scale ranging from 0 to 14 points—with 0 indicating normal neurological function and 14 representing the most severe deficits (23). The score integrates assessments of motor function, balance, and reflexes; one point was awarded for the inability to complete a test or the absence of a tested reflex, such that higher scores corresponded to more severe neurological injury.

Testing components and scoring criteria were as follows:

- Tail suspension test (3 points total): 1 point each for forelimb flexion, hindlimb flexion, or head deviation >10°from the vertical axis within 30 seconds.
- Floor walking test (3 points total): 0 points for normal walking; 1 point for inability to walk straight; 2 points for circling toward the paretic side; 3 points for falling toward the paretic side.
- Beam balance test (6 points total): 0 points for steady balance; 1 point for grasping the beam’s side; 2 points for hugging the beam with one limb falling off; 3 points for hugging the beam with two limbs falling off or spinning on the beam (>30 seconds); 4 points for attempting to balance but falling off (>20 seconds); 5 points for attempting to balance but falling off (>10 seconds); 6 points for falling off without attempting to balance or hang on (<10 seconds).
- Reflex assessment (2 points total): 1 point each for the absence of the pinna reflex (no head shake when touching the auditory meatus) or corneal reflex (no eye blink when lightly touching the cornea with cotton).

Foot-Fault Test: Mice were placed on a wire grid and recorded for 3 minutes. A foot fault was defined as a misplacement of the right forelimb or hindlimb through the grid. The fault rate (%) was calculated as (fault steps/total steps) × 100.

Adhesive Removal Test: To assess sensorimotor deficits, a 3×3 mm adhesive tape was affixed to the plantar surface of the forepaw. The time taken for the mouse to first sense (sensory latency) and completely remove the tape (removal time) was recorded, with a maximum cutoff of 2 minutes. Three trials were performed at 10-minute intervals, and the average latency and removal time were calculated. Prolonged latency or removal time indicates impaired somatosensory function.

TTC Staining: At 72 h post-tMCAO, brains were harvested, coronally sectioned (1 mm thickness), and incubated in 1% 2,3,5-triphenyltetrazolium chloride (TTC; Sigma-Aldrich, T8877) at 37 °C for 30 min. After fixation with 4% paraformaldehyde, unstained pale regions were identified as infarct areas.

Determination of stroke size (24): Infarct volume percentage was quantified in ImageJ by comparing intact ipsilateral versus contralateral hemispheric volumes across five consecutive sections (prepared as described in the TTC Staining section above).

Edema - corrected infarct volumes were calculated via planimetry (ImageJ software, NIH) using the formula (as referenced in 24):


$${\text{V}}_{\text{infarct}}(\%)\;={\text{ V}}_{\text{infarct}}/{\text{V}}_{\text{C}}\times \text{ (}1-({\text{V}}_{\text{I}}{\text{– V}}_{\text{C}})/{\text{V}}_{\text{C}})$$

V_I_ = volume of the ischemic (ipsilateral) hemisphere.
V_C_ = volume of the non - ischemic (contralateral) hemisphere.

This equation adjusts for edema by normalizing the infarct volume to the contralateral hemisphere, ensuring accurate quantification of tissue damage independent of post - ischemic swelling.

Fluoro-Jade C (FJC) Staining for Degenerating Neuron Quantification: Perfusion-fixed brains were cryosectioned into 20 μm coronal sections. Sections were incubated with anti-NeuN antibody (Abcam, ab177487, 1:1000) at 4 °C for 24 h, washed three times with PBS, then stained with freshly prepared Fluoro-Jade C solution (Biosensis, TR-100-FJ) for 10 min in the dark, with distilled water washes between steps. Sections were counterstained with DAPI, cleared in xylene, and mounted with DPX medium for imaging. Five standardized, non-overlapping 200 × 200 μm regions of interest (ROIs) were randomly selected in the peri-infarct region per brain section, with consistent ROI area across all samples. FJC^+^NeuN^+^ degenerating neurons within each ROI were counted via ImageJ.

### 2.3 Molecular biology assays

Cytokine Analysis: Ischemic brain hemispheres (n=4/group) were lysed in RIPA buffer containing protease inhibitors (Sigma, P2714) at 3 days post-tMCAO. Csf3 (Proteintech, KE10025), IL-1b (ExCell Bio, EM001), IL-6 (ExCell Bio, EM004), and Tnfα (ExCell Bio, EM008) levels were quantified using species-specific ELISA kits following manufacturer protocols.

qPCR: Total RNA was isolated with TRIzol (Invitrogen, 15596026) and reverse-transcribed (Takara, RR047A). SYBR Green-based qPCR (Takara, RR430A) was performed on a BioRad Opticon 2 system. Relative mRNA expression was calculated using the 2^−ΔΔCt^ method with *β*-actin normalization. Primers are listed in **Supplementary Table 2**.

Western Blot: Ischemic brain lysates (RIPA buffer, Beyotime, P0013B) were quantified by BCA assay (Beyotime, P0010S). Proteins (40 μg/lane) were separated by SDS-PAGE, transferred to PVDF membranes (Millipore), and probed with: Anti-Csf3 (Abcam, ab181053; 1:1000), Anti-IL1b (Affinity, AF5103; 1:1000), Anti-IL6 (Affinity, DF6087; 1:1000), Anti-Tnfα (Affinity, AF7014; 1:1000), Anti-β-tubulin (Cell Signaling, 2146; 1:1000), HRP-conjugated secondary antibodies (R&D Systems; 1:1000) were detected by chemiluminescence (Millipore), quantified using ImageJ (n=4/group).

Immunofluorescence Staining: Perfusion-fixed brains were cryosectioned at 20 μm thickness. Sections underwent sequential incubation with primary antibodies: anti-Iba1 (Wako, #011-27991; 1:300), anti-CD86 (Proteintech, #13395-1-AP; 1:300), followed by species-matched Alexa Fluor-conjugated secondary antibodies (Thermo Fisher; 1:1000). Quantification of Iba1^+^CD86^+^ dual-positive cells was performed in five non-overlapping 200 × 200 μm microscopic fields in peri-infarct regions. Blinded evaluators performed cell counts using ImageJ, applying co-localization criteria (>50% signal overlap).

### 2.4 Transcriptomics and bioinformatics

RNA Sequencing: Total RNA from ischemic hemispheres (n=4/group) was isolated using Magzol Reagent (Magen) and quantified via K5500 spectrophotometer (Kaiao) and Agilent 2200 TapeStation. Poly(A) + mRNA was enriched using NEBNext^®^ Poly(A) mRNA Magnetic Isolation Module (NEB), fragmented to ~200 bp, and processed with NEBNext^®^ Ultra™ RNA Library Prep Kit for Illumina. Libraries were validated using Agilent 2200 TapeStation and Qubit (Thermo Fisher), followed by paired-end 150 bp sequencing on Illumina platforms (Ribobio). Differentially expressed genes (DEGs) were identified using DESeq2 (|log_2_FC|>0.58, *p* < 0.01).

GEO Cross-Analysis: The human stroke dataset GSE162955 (GPL17586 platform, Affymetrix HTA-2.0) was analyzed to identify differentially expressed genes (DEGs) between infarct core (IC) and contralateral healthy (CL) regions using R package. DEGs were defined as genes with *p* < 0.05. Intersecting DEGs between human and mouse datasets were visualized via Venn diagrams.

### 2.5 *In vitro* studies

Cell Culture and OGD/R Model: BV2 microglia (Procell Life Science) were cultured in high-glucose DMEM supplemented with 10% FBS and 1% penicillin/streptomycin under standard conditions (37 °C, 5% CO₂). For OGD/R modeling, cells (5×10³/well in 96-well plates) were exposed to glucose/serum-free DMEM in a hypoxic chamber (1% O₂/5% CO₂/94% N₂, 37 °C, 6 h) followed by 24 h reoxygenation in normal glucose DMEM with 10% FBS. Experimental groups included: Control: Untreated cells in PBS; Vehicle: OGD/R + PBS; LJP-treated: OGD/R + LJP (12.5–200 μM, administered 2 h pre-OGD and sustained during reoxygenation); LJP + Csf3: LJP with recombinant Csf3 (50 ng/mL during reoxygenation, PeproTech, 250-05); LJP + Anti-Csf3: LJP with Csf3-neutralizing antibody (4 μg/mL, R&mD Systems, MAB414).

Cell Viability Assay: After reoxygenation, 10 μL CCK-8 reagent was added to each well and incubated for 2 h. Absorbance was measured at 450 nm using a microplate reader.

Reactive Oxygen Species (ROS) Detection: Cells were incubated with 10 μM DCFH-DA (Beyotime, China) for 30 min. Fluorescence intensity was quantified at Ex/Em 488/525 nm using a confocal microscope.

Immunofluorescence Staining: Fix the cells with 4% PFA at room temperature for 15 minutes. Use rabbit anti - Csf3 (abcam; ab181053; 1:400) as the primary antibody. Visualize the signal with fluorescence Alexa Fluor 555 secondary antibodies (1:1000; Thermo Fisher). Capture images of the cells using a confocal microscope and then process all the images with ImageJ software.

### 2.6 Statistical analysis

Data were presented as scattered dot plots, mean ± SD. Statistical comparisons between two groups were performed using Student’s t-test. For multi-group analyses, one-way or two-way ANOVA (parametric) was applied, followed by Tukey’s *post hoc* test for pairwise comparisons. All analyses were conducted with GraphPad Prism 9.0 (GraphPad Software, USA), and statistical significance was defined as *P* < 0.05.

## 3 Results

### 3.1 Preparation and structural characterization of LJP

**Figure 1:**
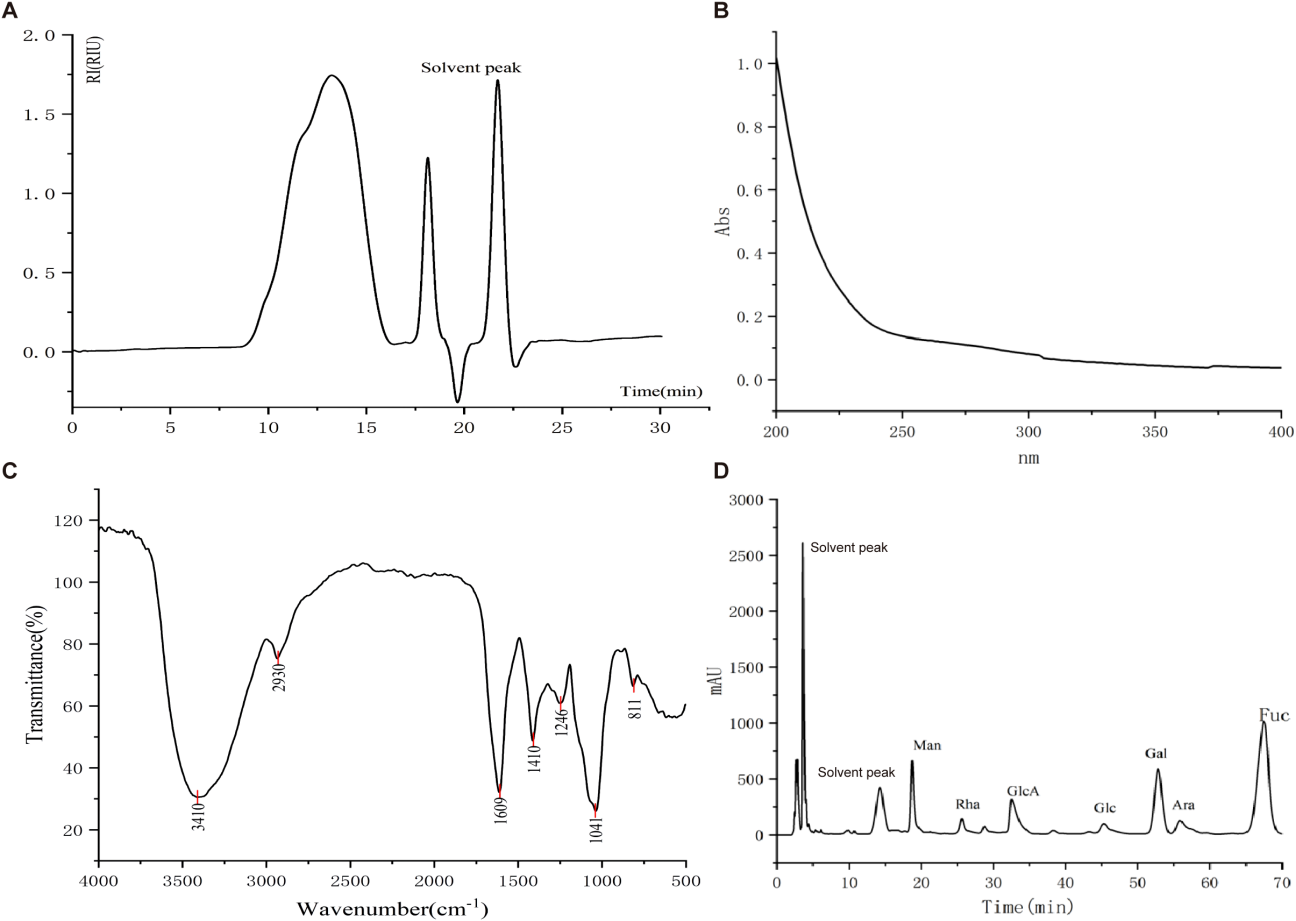
Structural characterization of Laminaria japonica polysaccharide (LJP). **(A)** HPGPC profile showing the major molecular weight peaks of LJP (264,241 Da). **(B)** UV absorption spectrum of LJP (200–400 nm), confirming the absence of protein/nucleic acid contamination. **(C)** FT-IR spectra identifying functional groups: O–H (3410 cm^-1^), C=O (1609 cm^-1^), and S=O (1246 cm^-1^). **(D)** Ion chromatography (IC) analysis of monosaccharide composition.

LJP was isolated via a standardized protocol involving hot water extraction, sequential ethanol precipitation, and deproteinization to yield a purified fraction. High-performance gel permeation chromatography (HPGPC) analysis identified a major molecular weight component of 264,241 Da, indicating relative homogeneity of the isolated polysaccharide (**Figure 1A**). Quantitative analyses revealed that LJP contained 70.12% total sugar, 22.03% sulfate groups, and 7.81% uronic acid—key structural features implicated in the bioactivity of sulfated polysaccharides. Ultraviolet (UV) spectroscopy (200–400 nm) showed no detectable absorption at 260 nm (nucleic acid marker) or 280 nm (protein marker), confirming minimal contamination by nucleic acids or proteins (**Figure 1B**). Fourier-transform infrared (FT-IR) spectroscopy further validated LJP as a sulfated polysaccharide, with characteristic peaks corresponding to O–H stretching vibrations (3410 cm^-1^), carbonyl (C=O) groups (1609 cm^-1^), and sulfate ester (S=O) linkages (1246 cm^-1^) (**Figure 1C**). Ion chromatography (IC) was used to determine the monosaccharide composition, revealing that LJP consists of fucose (Fuc), galacturonic acid (GalA), glucuronic acid (GlcA), mannose (Man), and arabinose (Ara) in a molar ratio of 0.506:0.204:0.106:0.088:0.045 (**Figure 1D**; **Supplementary Table 3**). Notably, the monosaccharide profile of our isolated LJP exhibits subtle discrepancies compared to previously reported compositions (22), which may be attributed to variations in seaweed harvesting location, growth conditions, or extraction parameters.

### 3.2 LJP improves neurological outcomes and reduces infarct volume

**Figure 2:**
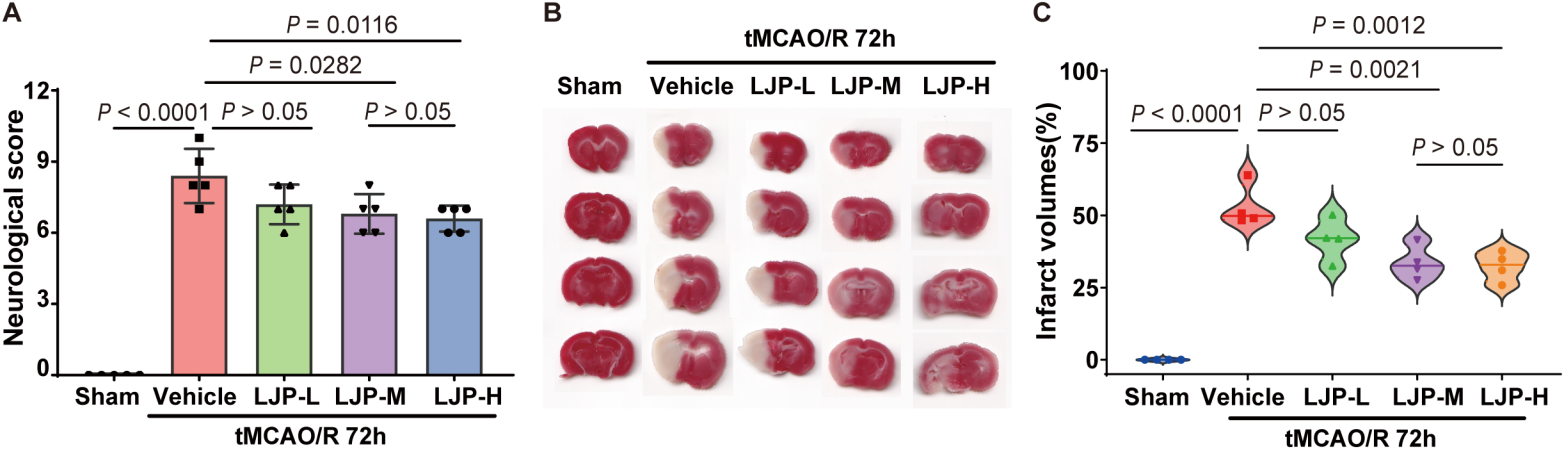
LJP improves neurological function and reduces infarct volume in tMCAO mice. **(A)** Modified Neurological Severity Score (mNSS) at 72 h post-I/R in the Sham, Vehicle, LJP-L (100 mg/kg), LJP-M (200 mg/kg) and LJP-H (400 mg/kg) groups. **(B)** Representative TTC-stained brain sections. **(C)** Quantification of infarct volume.

To evaluate the neuroprotective effects of LJP, behavioral assessments were conducted 72 hours post-tMCAO induction. LJP-M (200 mg/kg) and LJP-H (400 mg/kg) treated animals exhibited significant improvements in neurological function, as demonstrated by markedly reduced modified Neurological Severity Scores (mNSS) compared to vehicle controls (**Figure 2A**). While LJP-L (100 mg/kg) trended toward lower mNSS values relative to vehicle, this improvement did not reach statistical significance. TTC staining further revealed a substantial attenuation of ischemic brain injury, both LJP-M and LJP-H significantly reduced infarct volume compared to vehicle (**Figures 2B, C**). No significant difference was observed between LJP-M and LJP-H groups, indicating a plateau in neuroprotective efficacy at doses ≥200 mg/kg. This plateau effect, common to complex polysaccharides, informed our selection of LJP-M for subsequent experiments.

### 3.3 LJP suppresses pro-inflammatory microglial polarization and inflammatory cytokine release

**Figure 3:**
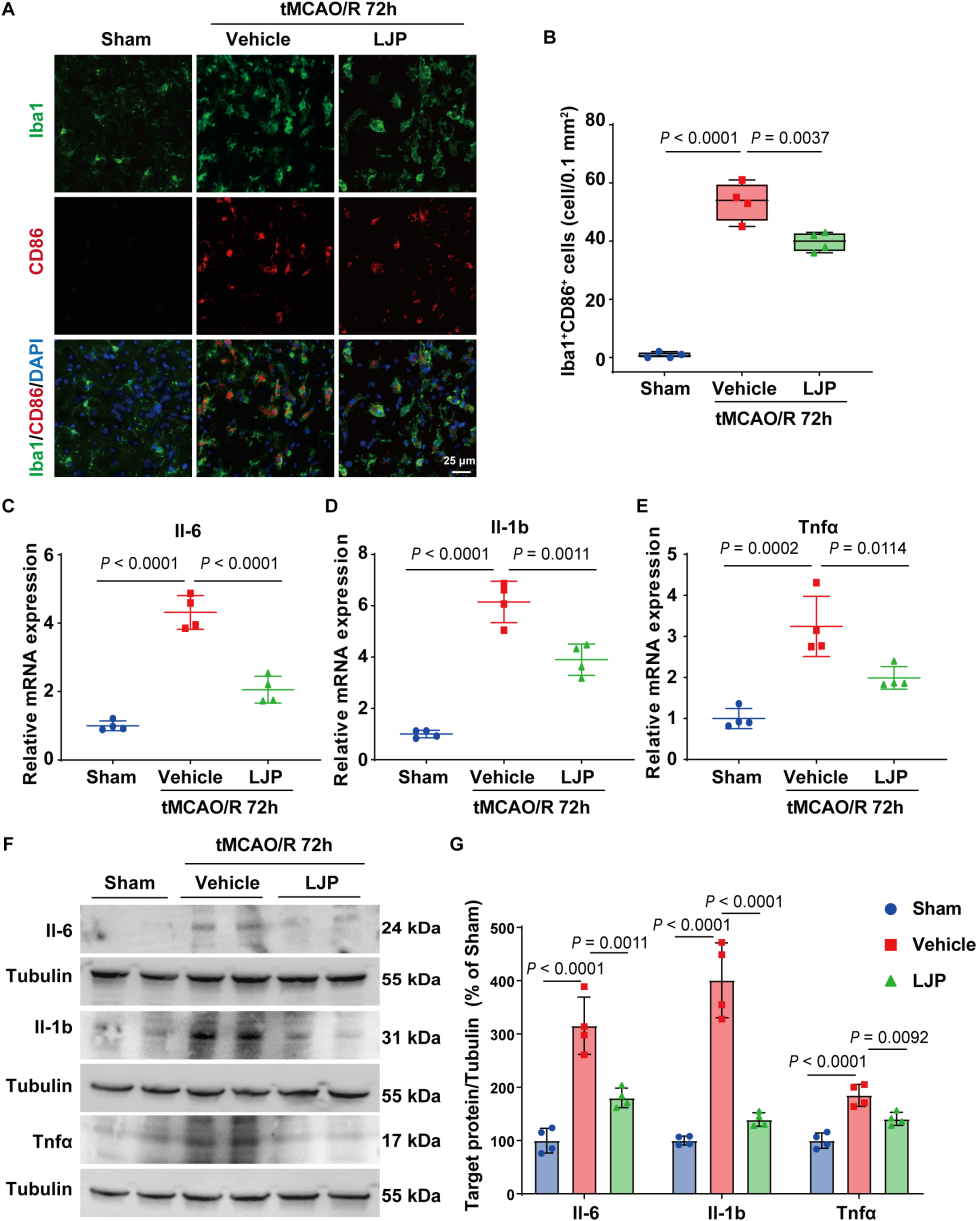
LJP suppresses pro-inflammatory microglial and pro-inflammatory cytokine release. **(A)** Immunofluorescence co-staining of Iba1 (green) and CD86 (red) in peri-infarct cortex (CD86 marks pro-inflammatory microglia). **(B)** Quantification of Iba1^+^/CD86^+^ cells. **(C–G)**. qPCR and western blots show the expression of IL-1b, Tnfα, and IL-6 in the ischemic brain tissues at 72 h after tMCAO/R.

Microglia, the primary innate immune cells in the brain, play a pivotal role in post-stroke neuroinflammation. Immunofluorescence co-staining demonstrated that LJP treatment induced a significant suppression of microglial polarization toward an pro-inflammatory phenotype, as evidenced by reduced colocalization of Iba1^+^CD86^+^ cells (a marker of pro-inflammatory microglia) compared to vehicle controls (**Figures 3A, B**). In line with this, LJP treatment also reduced the expression of iNOS, another key marker of pro-inflammatory microglia, while increasing the expression of CD206, a canonical marker of anti-inflammatory/regulatory microglia (**Supplementary Figure 1**). Molecular profiling via qPCR and immunoblotting further revealed that LJP administration robustly attenuated the expression of pro-inflammatory mediators, including IL-6, IL-1b, and Tnfα, in the ischemic brain tissues (**Figures 3C–G**). Collectively, these findings establish that LJP mitigates cerebral I/R injury by suppressing pro-inflammatory microglial polarization and inflammatory cytokine release.

### 3.4 Csf3 contributes to LJP’s neuroprotective effects

**Figure 4:**
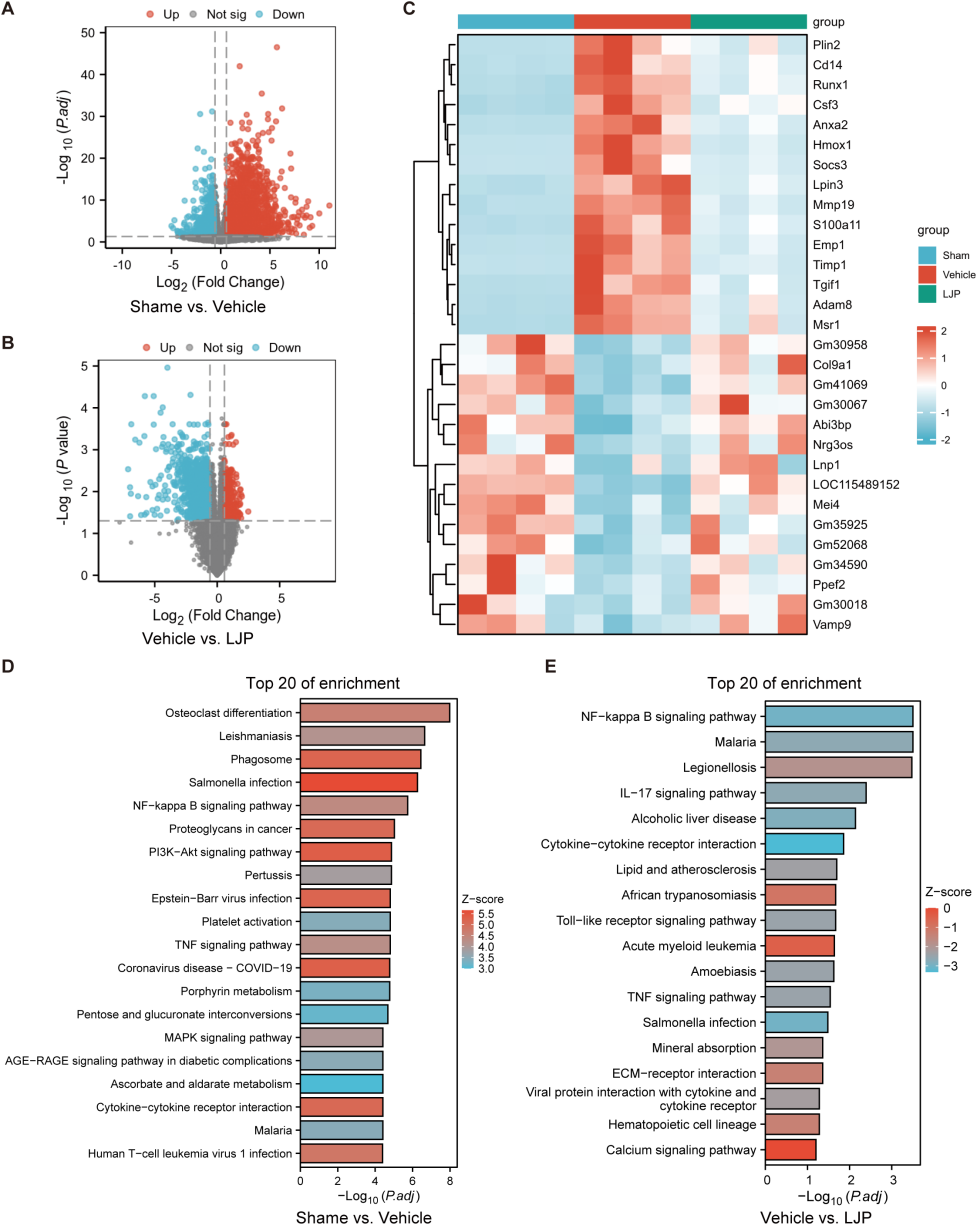
Transcriptomic profiling identifies pro-inflammatory pathway dysregulation in ischemic brains and modulation by LJP. A-C. Volcano plots illustrating differentially expressed genes (DEGs) between Sham *vs*. Vehicle groups **(A)** and Vehicle *vs*. LJP groups **(B)**. Heatmap **(C)** shows hierarchical clustering of DEGs across all experimental groups, reflecting distinct transcriptional signatures associated with ischemia and LJP treatment. D-E. KEGG pathway enrichment histogram displaying the top 20 enriched pathways: **(D)** Pathways enriched in Vehicle *vs*. Sham groups (positive z-scores, 3-5; *P <* 0.01), highlighting activated pro-inflammatory signaling networks post-ischemia. **(E)** Pathways enriched in LJP *vs*. Vehicle groups (negative z-scores, -3 to 0; *P <* 0.01), indicating LJP-mediated suppression of pro-inflammatory pathways.

To elucidate the molecular mechanisms underlying LJP’s anti-inflammatory actions, we performed transcriptome sequencing on ischemic brain tissues from Sham, Vehicle, and LJP-treated mice (**Figures 4A–C**; **Supplementary Table 4** and **S5**). Comparative analysis between the Sham and Vehicle groups identified differentially expressed genes (DEGs) linked to post-ischemic pathological processes. KEGG pathway enrichment analysis integrated with logFC values revealed the top 20 significantly enriched pathways, with prominent activation of key inflammatory signaling cascades including the NF-kappa B signaling pathway, TNF signaling pathway, MAPK signaling pathway, and cytokine-cytokine receptor interaction (**Figure 4D**).

In contrast, KEGG enrichment analysis of DEGs between the Vehicle and LJP-treated groups yielded the top 20 pathways with negative z-scores, indicating LJP-mediated suppression of these pro-inflammatory signaling networks (**Figure 4E**). Notably, LJP specifically downregulated multiple pathways that were hyperactivated in the Vehicle group, most notably the NF-kappa B signaling pathway, TNF signaling pathway, cytokine-cytokine receptor interaction, and Toll-like receptor signaling pathway. This transcriptional signature of reduced pro-inflammatory pathway activity dovetails with our earlier functional observations that LJP suppresses microglial polarization toward a pro-inflammatory phenotype and reduces pro-inflammatory cytokine production (**Figure 3**). Together, these findings reinforce that LJP exerts anti-inflammatory effects at both the functional and transcriptional levels, with pathway-level suppression providing a molecular framework for the observed phenotypic changes.

**Figure 5:**
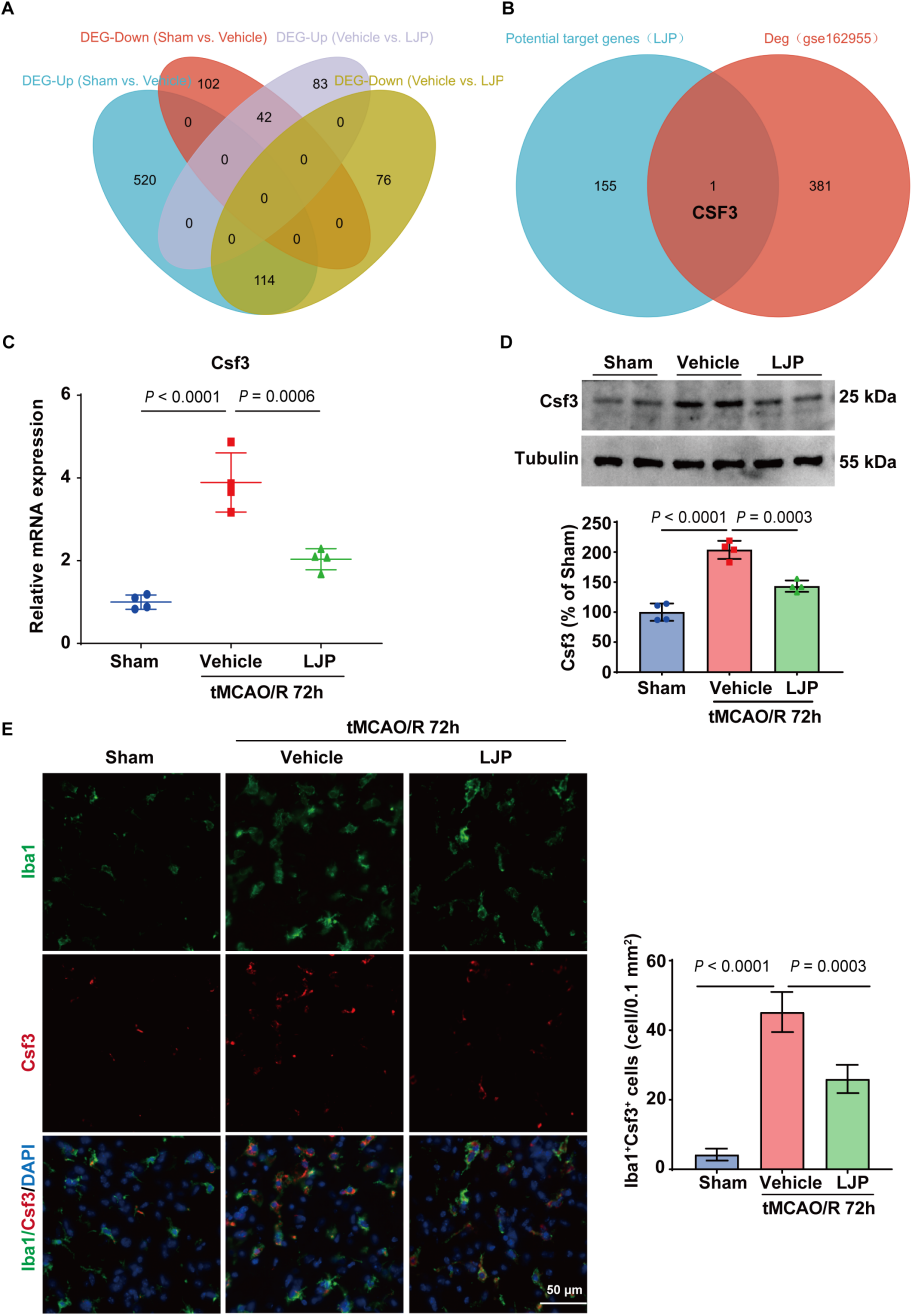
Csf3 is a conserved, LJP-regulated potential target linked to neuroprotection. **(A)** Venn diagram depicting the overlap of DEGs from Sham *vs*. Vehicle and Vehicle *vs*. LJP comparisons, identifying 156 genes potentially associated with LJP’s neuroprotective effects. **(B)** Venn diagram illustrating Csf3 as the sole overlapping gene between murine tMCAO DEGs and human stroke transcriptomic data (GEO: GSE162955), confirming its evolutionary conservation across species. C–D. Validation of Csf3 expression by qPCR **(C)** and western blot **(D)** in ischemic brain tissues at 72 h post-tMCAO/R. **(E)** Immunofluorescence co-staining of Iba1 (green, microglial marker) and CSF3 (red) in peri-infarct regions.

Comparative analysis identified 156 differentially expressed genes (DEGs) potentially linked to LJP-mediated neuroprotection (**Figure 5A**). To identify evolutionarily conserved, clinically relevant gene targets, these mouse-derived neuroprotection-associated DEGs were then cross-referenced with pathogenic DEGs identified in human stroke brain tissue (GEO: GSE162955); this sequential, predefined analysis revealed Csf3 as the sole overlapping gene between the two curated gene sets (**Figure 5B**). Csf3 expression was significantly upregulated in both tMCAO mice and human stroke brains, demonstrating diagnostic relevance (**Figures 5C, D**; **Supplementary Figure 2**, **S3**). Importantly, LJP administration suppressed Csf3 transcription and translation while reducing Csf3 immunoreactivity in IBA-1-positive microglia within injured brain regions (**Figures 5C–E**).

These findings implicate Csf3 as a potential mechanistic node in LJP’s neuroprotective effects. However, we recognize that transcriptomic overlap alone cannot establish causality, and that complex natural products typically modulate multiple targets. Subsequent functional studies were therefore designed to test whether Csf3 modulation represents a necessary, though perhaps not sufficient, component of LJP’s therapeutic mechanism.

### 3.5 LJP attenuates OGD/R-induced microglial inflammation via Csf3 suppression

**Figure 6:**
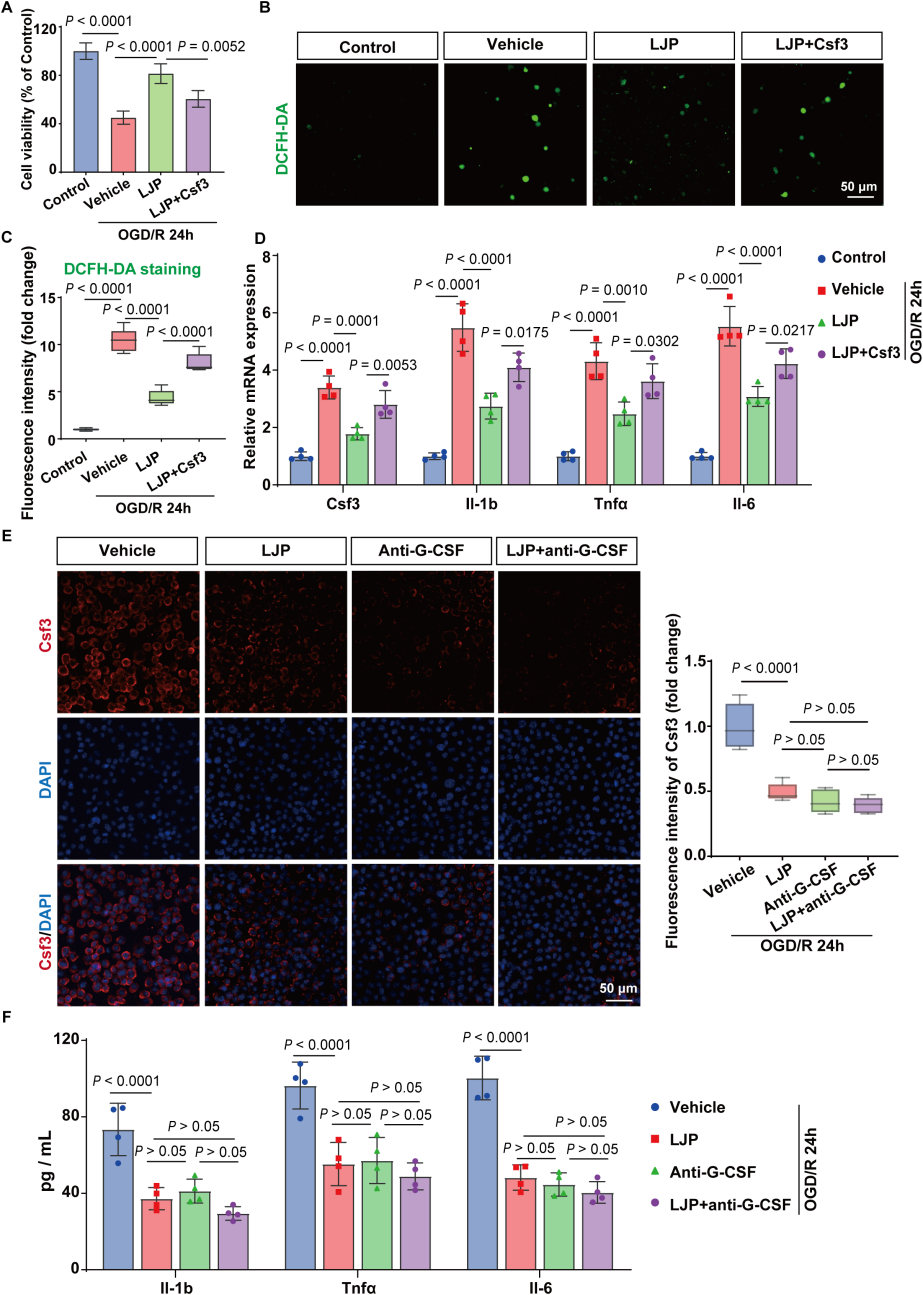
LJP attenuates OGD/R-induced inflammation in BV2 microglia via Csf3. **(A)** CCK-8 assay of cell viability. **(B, C)**. Intracellular ROS levels were measured by DCFH-DA fluorescence. **(D)** qPCR shows the expression of Csf3, IL-1b, Tnfα and IL-6 in OGD/R-stimulated BV2 cells. **(E)** Representative immunofluorescence images of Csf3 (red) and DAPI (blue) in BV2 cells. **(F)** ELISA present the levels of IL-1b, Tnfα and IL-6 were detected in the supernatant of OGD/R-stimulated BV2 cells (n = 4).

To evaluate LJP’s anti-inflammatory effects in cerebral I/R injury, we subjected BV2 microglia to oxygen-glucose deprivation/reoxygenation (OGD/R). CCK-8 assays showed that LJP treatment (25, 50, 100 μM) dose-dependently restored cell viability post-OGD/R, with maximal protection observed at 100 μM (selected for subsequent experiments; **Figure 6A**; **Supplementary Figure 4A**).

OGD/R induced a marked surge in intracellular ROS levels, a key marker of oxidative stress, which was attenuated by LJP (**Figures 6B, C**). qPCR and ELISA analyses revealed that LJP significantly suppressed OGD/R-induced upregulation of pro-inflammatory mediators, including Csf3, IL-1b, Tnfα, and IL-6 (**Figure 6D**, **Supplementary Figure 4B**).

Notably, recombinant Csf3 administration partially reversed LJP’s protective effects, diminishing cell viability restoration, increasing ROS production, and restoring cytokine secretion (**Figures 6A–D**). Furthermore, LJP combined with a Csf3-neutralizing antibody showed no additive anti-inflammatory benefits compared to LJP alone (**Figures 6E, F**). These observations indicate that LJP’s effects involve Csf3-mediated pathways, though the incomplete reversal suggests additional mechanisms likely contribute. The lack of enhanced efficacy with Csf3 neutralization further implies that LJP already optimally suppresses Csf3-dependent signaling in this context.

### 3.6 LJP alleviates post-stroke neuroinflammation via Csf3 inhibition

**Figure 7:**
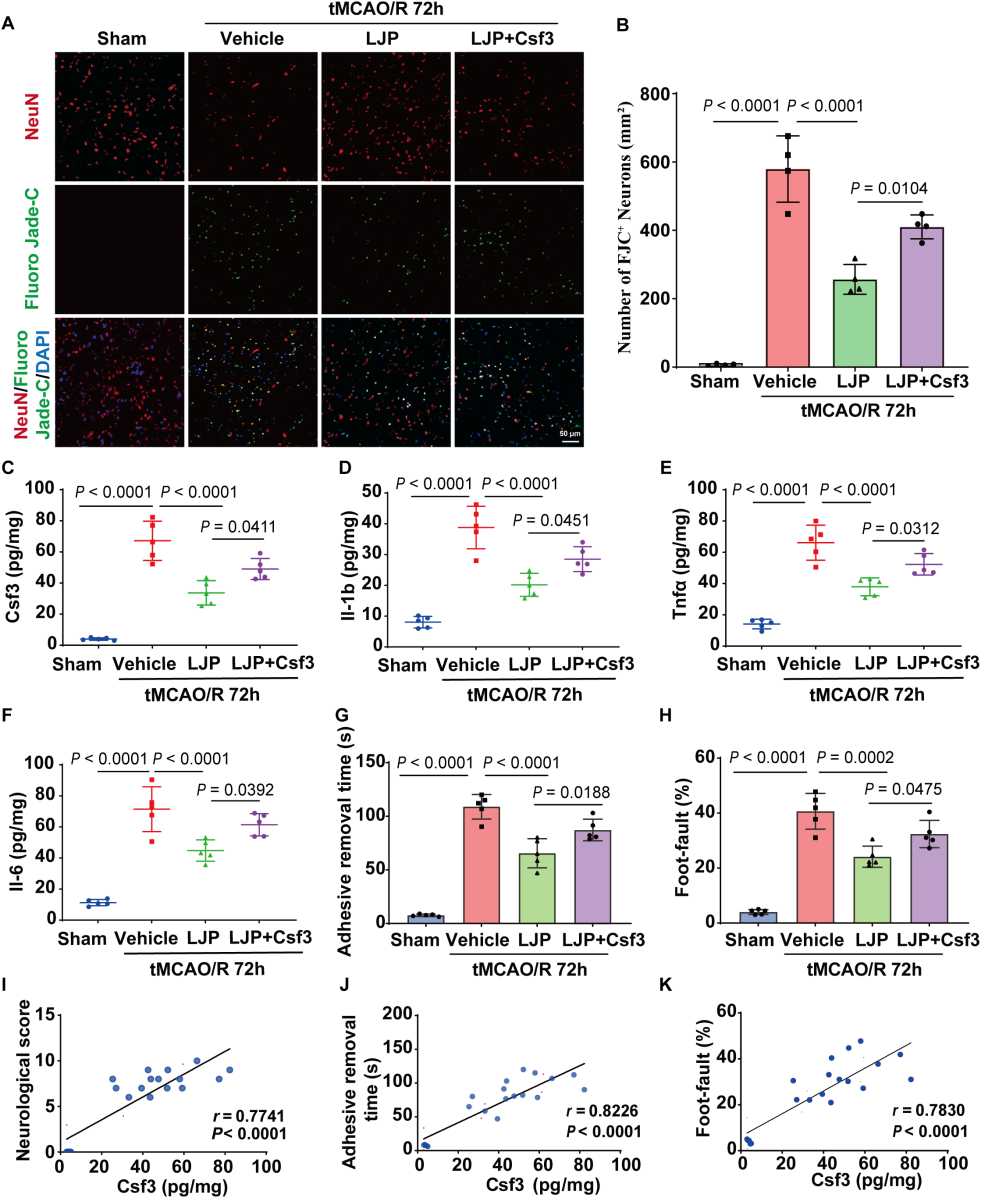
Csf3 mediates LJP’s neuroprotective effects *in vivo*. Representative images of the co-localization of FJC (green) staining in peri-infarct cortex at 72 h after tMCAO/R. **(B)** Quantitative analyses of FJC-positive cells in the peri−infarct area at 72 h after tMCAO/R (n = 4). **(C–F)** ELISA present the expression of Csf3, IL-1b, Tnfα and IL-6 in the ischemic brain (n = 5). **(G, H)** Behavior tests are performed 72 h after injury, including adhesive removal time **(G)** and foot fault tests **(H)**. **(I–K)** Correlation analysis between Csf3 expression and neurological deficits.

Fluoro-Jade C (FJC) staining demonstrated that LJP treatment significantly reduced neurodegeneration in peri-infarct cortex at 72 h post-I/R (**Figures 7A, B**). Concurrently, LJP downregulated cerebral levels of Csf3, IL-1b, IL-6, and Tnfα compared to vehicle-treated mice (**Figures 7C–F**). Behavioral assessments corroborated these findings: LJP reduced adhesive removal time and decreased foot-fault frequency (**Figures 7G, H**). Strikingly, recombinant Csf3 administration partially reversed LJP’s therapeutic effects, increasing neurodegeneration, restoring cytokine secretion, increasing adhesive removal time and foot-fault frequency (**Figures 7A–H**).

Correlation analyses revealed strong positive associations between Csf3 expression and neurological impairment: mNSS (*r* = 0.77), adhesive removal time (*r* = 0.82), and foot-faults (*r* = 0.78) (**Figures 7I–K**). These data confirm that LJP mitigates post-I/R neuroinflammation and functional deficits at least partly through Csf3 modulation.

## 4 Discussion

This study explores Laminaria japonica polysaccharide (LJP)—a sulfated heteropolysaccharide enriched in fucose, galacturonic acid, glucuronic acid, and sulfate esters—as a candidate agent for cerebral ischemia/reperfusion (I/R) injury, with mechanistic evidence linking its neuroprotective effects to Csf3 signaling modulation. Three key observations emerged: (1) Structural characterization highlights LJP’s sulfate-rich composition, a structural trait widely linked to the immunomodulatory activity of marine sulfated polysaccharides; (2) Cross-species multi-omics integration identifies Csf3—a cytokine linked to microglial polarization toward a pro-inflammatory phenotype—as a potential LJP-regulated target; (3) LJP suppresses Csf3-driven neuroinflammation, while concurrently attenuating oxidative stress and neuronal apoptosis. Notably, this work defines a discrete mechanistic link between LJP and Csf3 signaling in cerebral I/R injury, a regulatory axis not fully defined in previous fucoidan-focused studies. Together, these findings support LJP’s potential as a multimodal phytotherapeutic candidate for ischemic stroke.

LJP, particularly fractions rich in sulfate groups, has well-documented antioxidant activity in the literature—an attribute closely tied to its structural features. Li et al. (9) noted that sulfate moieties in LJP directly scavenge reactive oxygen species (ROS), such as hydroxyl and superoxide radicals, and inhibit lipid peroxidation—key processes for mitigating oxidative stress in cerebral I/R injury. Complementing this, Li et al. (25) further demonstrated that LJP reduces intracellular ROS levels in a dose-dependent manner in oxidative stress models—findings that similar with our *in vitro* observations of LJP-mediated ROS suppression in OGD/R-challenged BV2 microglia.

Beyond antioxidant activity, the sulfate moieties and uronic acids in LJP may underpin its bioactivity. Sulfated polysaccharides are known to engage pattern recognition receptors (e.g., TLR4) through electrostatic interactions (9, 12), and our Fourier-transform infrared spectroscopy (FT-IR) and ion chromatography (IC) analyses confirmed the presence of these structural features in LJP. This raises the possibility that LJP may competitively inhibit TLR4/NF-κB signaling in microglia—an hypothesis that merits further investigation via molecular docking studies. Notably, LJP’s concurrent suppression of NF-κB and NLRP3 pathways (26–28), as evidenced by reduced IL-1β and TNF-α levels, suggests broad-spectrum immunomodulatory effects distinct from single-target synthetic agents.

Csf3 (G-CSF), traditionally recognized for its hematopoietic functions, exhibits context-dependent roles in ischemic stroke pathology (13–17). While some studies suggest exogenous Csf3 may confer neuroprotection through neutrophil mobilization and enhanced neurogenesis, conflicting evidence links Csf3 administration to exacerbated infarct volume and inflammatory injury (17, 18, 29, 30). Clinically, trials and meta-analyses have reported no functional recovery benefits with Csf3 (15), indicating that Csf3’s early anti-apoptotic effects may be offset by persistent microglial-driven neuroinflammation. Our multi-omics analysis revealed marked Csf3 upregulation in both tMCAO mice and human stroke samples (GSE162955), which correlated with worsened neurological outcomes. Critically, LJP treatment suppressed Csf3 transcription and translation, reducing Csf3 expression in IBA-1-positive microglia—accompanied by reduced microglial activation (fewer Iba1^+^CD86^+^ cells) and diminished IL-6/TNF-α release.

Mechanistically, emerging evidence highlights Csf3’s involvement in diverse inflammatory pathways. For instance, Csf3 exacerbates liver injury via LPS-TLR4 signaling in murine models, while its synergy with TLR4 inhibitors reduces inflammation and promotes tissue repair (31). Similarly, Csf3 inhibition mitigates acute lung injury by blocking MAPK/NF-κB activation (32). In stroke, endothelial CD36 amplifies neuroinflammation through Csf3-mediated neutrophil recruitment (16), and modulation of macrophage polarization by the Naoqing formula alleviates cerebral I/R injury via Csf3-related pathways (17). Intriguingly, our transcriptomic data align with these findings: LJP downregulated key inflammatory pathways, including the NF-κB signaling pathway, TNF signaling pathway, MAPK signaling pathway, and Toll-like receptor signaling pathway, in tMCAO/R mice. These observations suggest Csf3 may act as a nodal regulator of neuroinflammation and underscore LJP’s potential multimodal therapeutic effects.

While our data demonstrate that LJP suppresses Csf3 and that exogenous Csf3 can partially reverse LJP’s protective effects, several caveats warrant careful interpretation, as outlined below. First, the partial reversal observed in our rescue experiments—both *in vitro* and *in vivo*—provides compelling evidence that LJP engages multiple pathways beyond Csf3 modulation. This polypharmacology is characteristic of complex polysaccharides, which can simultaneously modulate TLR4/NF-κB, MAPK, and oxidative stress pathways, as supported by our transcriptomic analysis (**Figure 4E**). Notably, combined treatment with LJP and a Csf3-neutralizing antibody conferred no additional anti-inflammatory or neuroprotective benefits relative to LJP monotherapy. This observation is consistent with the possibility of saturable Csf3 signaling, as LJP mediates effective suppression of Csf3-dependent inflammatory cascades under our experimental conditions; thus, further antibody-mediated neutralization does not enhance therapeutic efficacy. Additionally, the partial reversal phenotype induced by exogenous recombinant Csf3 excludes the likelihood of redundant compensatory Csf3 pathways, as such redundancy would likely be associated with measurable additive effects following combined antibody treatment. Collectively, these data confirm that Csf3 signaling is a central, but not sole, mechanism underlying the neuroprotective actions of LJP. Second, while we have documented that exogenous recombinant Csf3 partially reverses LJP-mediated neuroprotection in our *in vivo* stroke model, formal causal validation of microglial Csf3 as a critical mediator remains incomplete, as we did not perform microglia-specific genetic loss-of-function studies (e.g., conditional knockout or targeted siRNA silencing) to directly confirm its *in vivo* role. Third, the current study lacks real-time cerebral blood flow monitoring, future studies will be needed to further characterize the effects of LJP on post-stroke cerebral perfusion to better clarify its neuroprotective mechanism. Fourth, the dichotomous role of Csf3 in stroke pathology remains debated: while some studies report neuroprotective effects through neutrophil mobilization, others demonstrate exacerbated inflammation. This context-dependency, paired with the lack of targeted genetic validation *in vivo*, underscores the need for microglia-specific genetic perturbation studies to more definitively establish the causal role of microglia-derived Csf3 in LJP’s mechanism of action. Future investigations should also evaluate whether LJP directly binds Csf3 or its receptors, or if Csf3 suppression occurs downstream of other primary targets, as well as confirm these mechanistic findings in refined, cell-type specific *in vivo* models.

## Data Availability

The original contributions presented in the study are publicly available. This data can be found at the NCBI Gene Expression Omnibus (GEO) under accession number GSE328360 (https://www.ncbi.nlm.nih.gov/geo/query/acc.cgi?acc=GSE328360).
