# Supplementary material for: Laminaria japonica polysaccharide mitigates acute neuroinflammation in cerebral ischemia-reperfusion injury through Csf3-modulated pathways
Source: Front Immunol. 2026 Apr 23;17:1801746. doi: 10.3389/fimmu.2026.1801746 (PMC13149078; doi:10.3389/fimmu.2026.1801746)
Supplement: Supplementary file 1 [file DataSheet1.zip › Supplementary Files/Supplemental figures.docx]

**Supplemental figures**


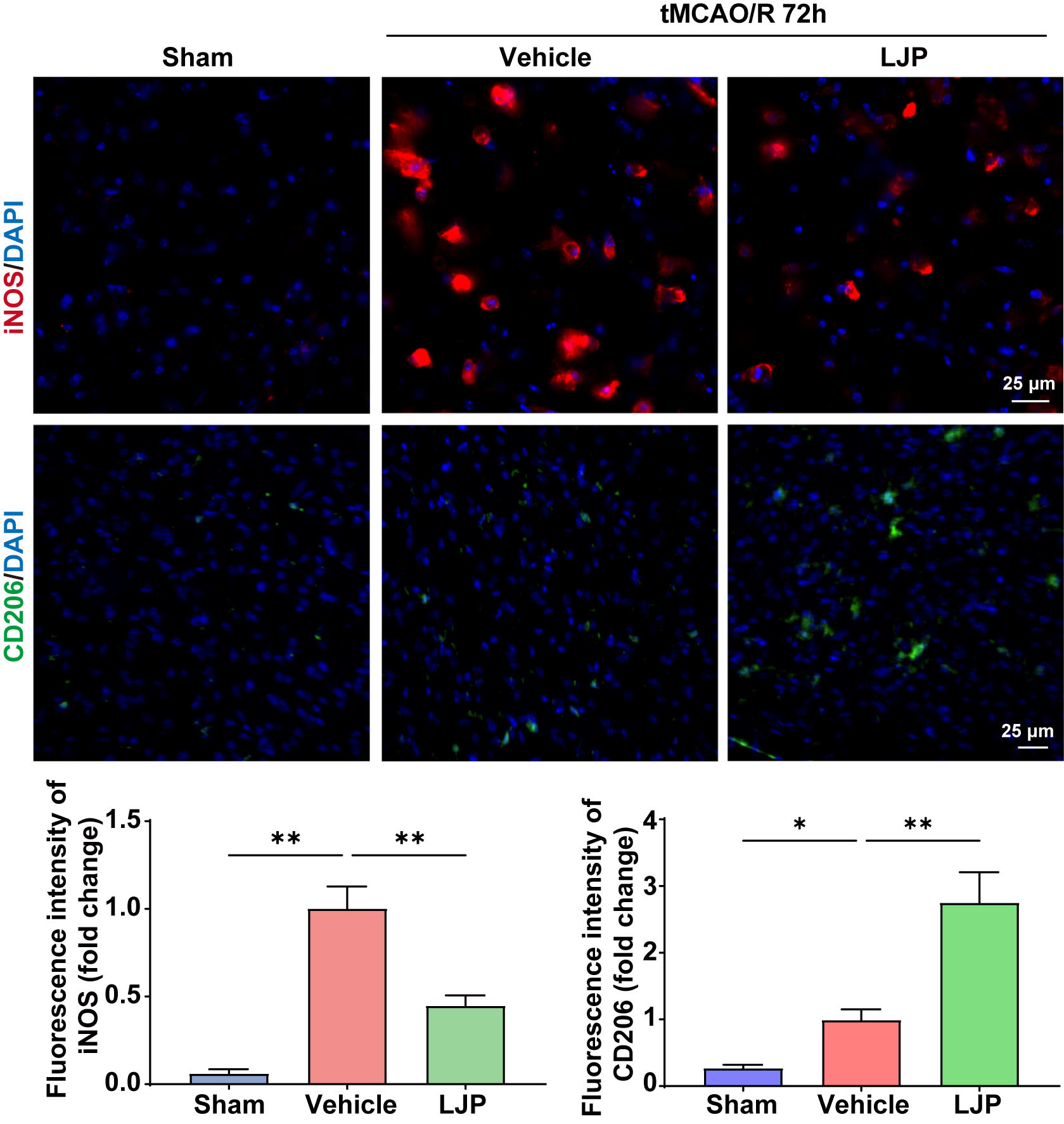


Figure S1. Immunofluorescence staining of iNOS and CD206 in peri-infarct cortex (n = 4). Data: mean ± SD; **p* < 0.05, ***p* < 0.01 vs. sham/vehicle.


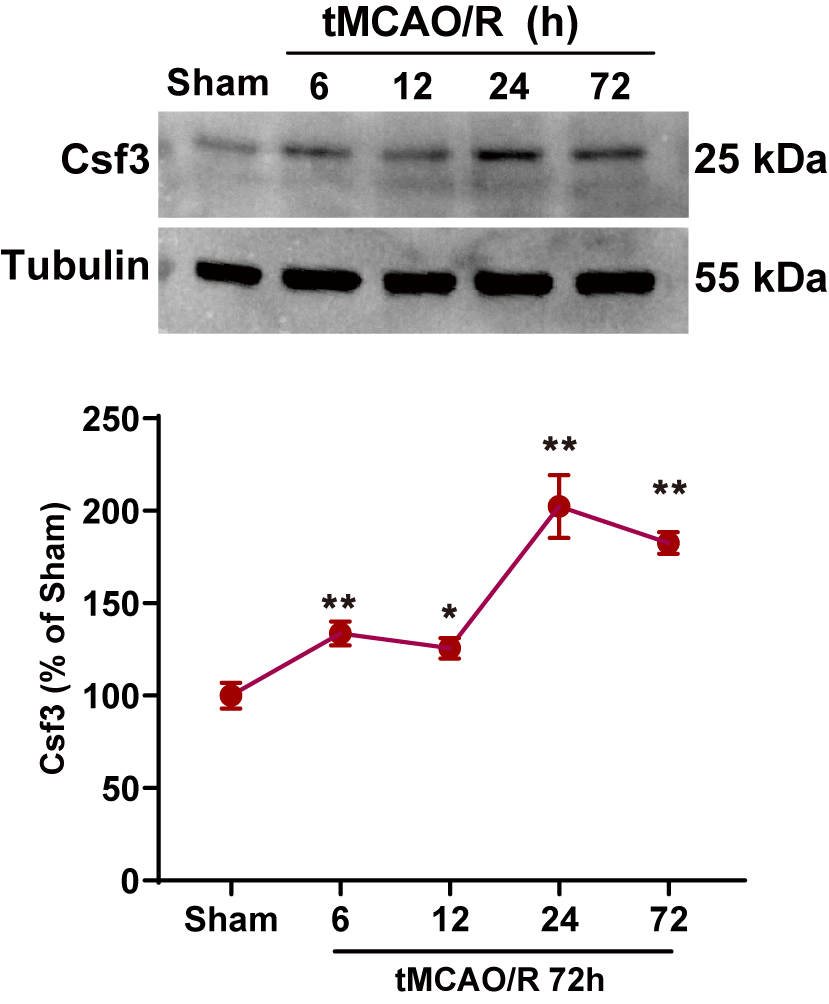


Figure S2. Western blots show the expression of Csf3 in the ischemic brain tissues at 6, 12, 24 and 72 h after tMCAO/R. **p* < 0.05, ***p* < 0.01 vs. Sham.


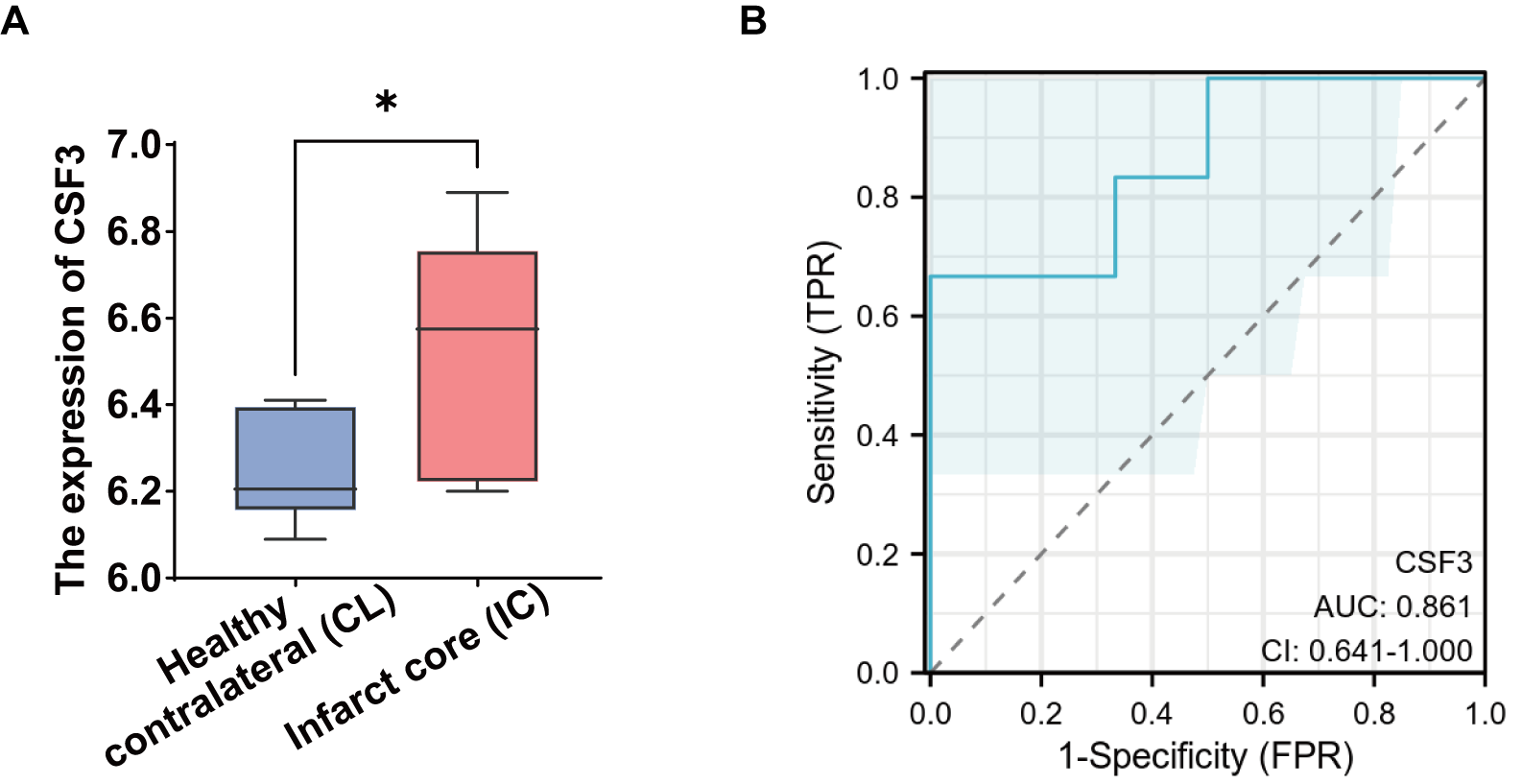


Figure S3. Elevated CSF3 is a sensitive marker of human ischemic stroke. A. Differential expression of CSF3 in brain pieces from the infarct core (IC) (GSE162955). B. ROC curves of CSF3 in stroke (GSE162955). Data: mean ± SD; **p* < 0.05 vs. CL.


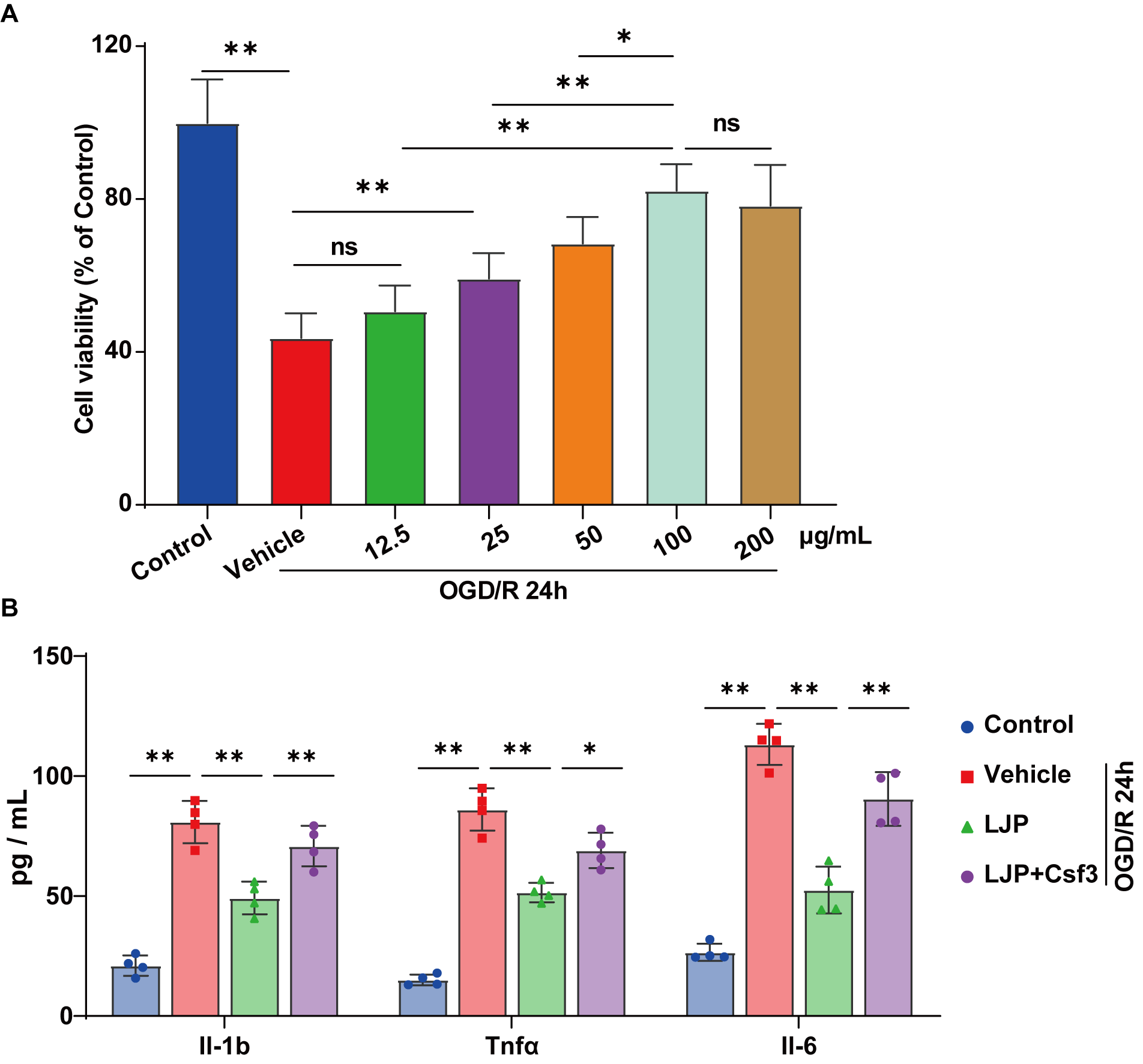


Figure S4. LJP attenuates OGD/R-induced inflammation in BV2 microglia via Csf3. A. CCK-8 assay of cell viability. B. ELISA present the levels of IL-1b, Tnfα and Il-6 were detected in the supernatant of OGD/R-stimulated BV2 cells (n = 4). Data: mean ± SD; **p* < 0.05, ***p* < 0.01 vs. sham/vehicle/LJP.


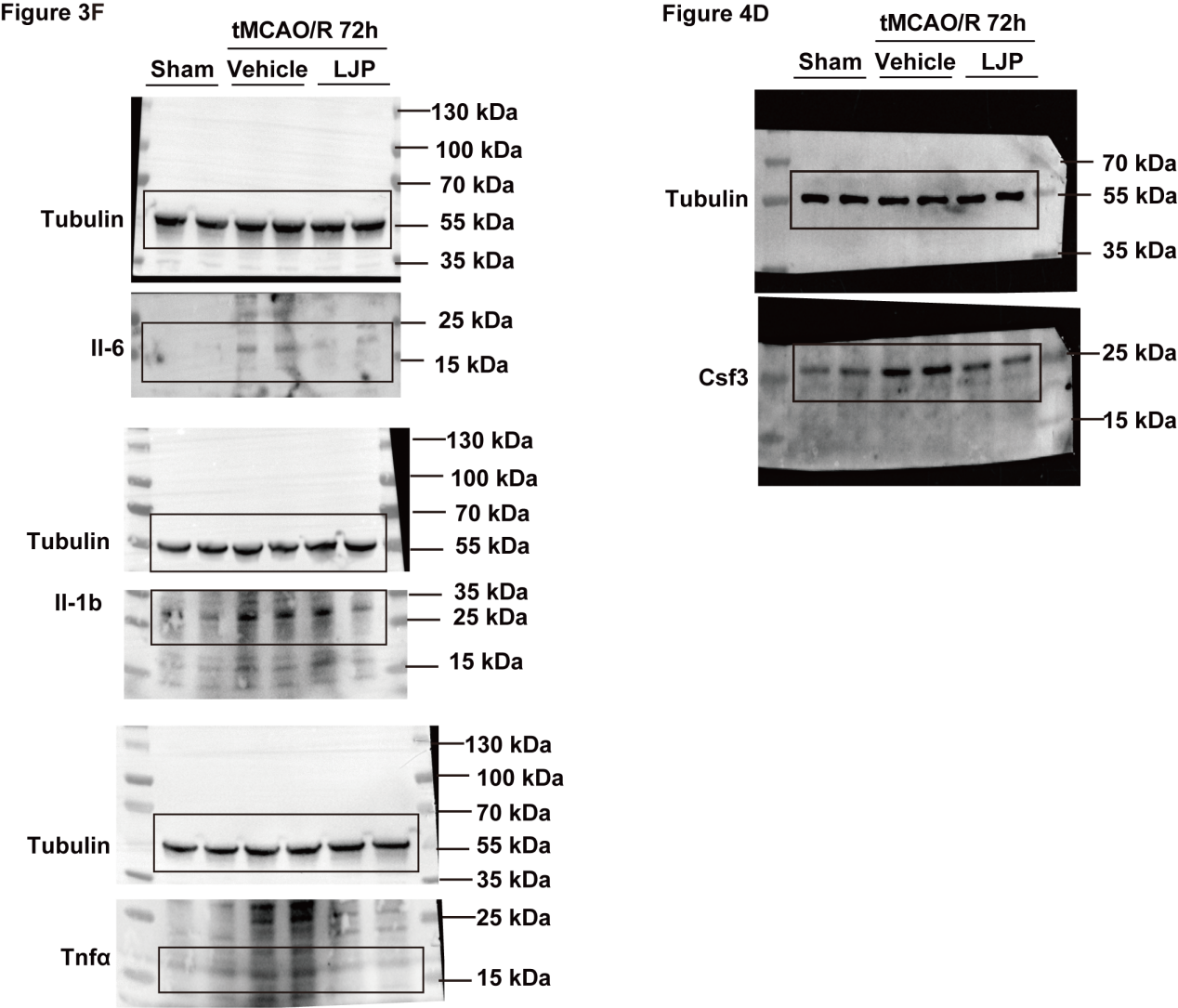


Figure S5. Original scan of the blots presented in the main text. Related to Figure 3F and Figure 4D.
