# Supplementary material for: Laminaria japonica polysaccharide mitigates acute neuroinflammation in cerebral ischemia-reperfusion injury through Csf3-modulated pathways
Source: Front Immunol. 2026 Apr 23;17:1801746. doi: 10.3389/fimmu.2026.1801746 (PMC13149078; doi:10.3389/fimmu.2026.1801746)
Supplement: Supplementary file 1 [file DataSheet1.zip › Supplementary Files/Table S1.DOCX]

**Table S1.** Experimental Groups

Experiment 1: Dose Optimization (40 mice total)

| Group | Initial n | Died Post - Surgery | Cerebral Hemorrhage | No Infarct (Excluded) | Final n (3 Days Post - tMCAO) |
| --- | --- | --- | --- | --- | --- |
| Sham | 8 | 0 | 0 | 0 | 8 |
| Vehicle | 8 | 2 | 1 | 0 | 5 |
| LJP-L | 8 | 2 | 1 | 0 | 5 |
| LJP-M | 8 | 1 | 0 | 1 | 6 |
| LJP-H | 8 | 1 | 0 | 1 | 6 |

Experiment 2: Confirmatory Study (36 mice total)

| Group | Initial n | Died Post - Surgery | Cerebral Hemorrhage | No Infarct (Excluded) | Final n (3 Days Post - tMCAO) |
| --- | --- | --- | --- | --- | --- |
| Sham | 10 | 0 | 0 | 0 | 10 |
| Vehicle | 13 | 3 | 1 | 0 | 9 |
| LJP-M | 13 | 2 | 1 | 0 | 10 |
