# Supplementary material for: Laminaria japonica polysaccharide mitigates acute neuroinflammation in cerebral ischemia-reperfusion injury through Csf3-modulated pathways
Source: Front Immunol. 2026 Apr 23;17:1801746. doi: 10.3389/fimmu.2026.1801746 (PMC13149078; doi:10.3389/fimmu.2026.1801746)
Supplement: Supplementary file 1 [file DataSheet1.zip › Supplementary Files/Table S2.DOCX]

Table S2. Primers used for qRT-PCR

| mRNA | Forward primer | Reverse primer |
| --- | --- | --- |
| *β*-actin | GGCTGTATTCCCCTCCATCG | CCAGTTGGTAACAATGCCATGT |
| Csf3 | AAGCTGTGTCACCCCGAGGAG | TGGAGCTGGCTTAGGCACTGT |
| Tnfα | CCCTCACACTCAGATCATCTTCT | GCTACGACGTGGGCTACAG |
| IL6 | TAGTCCTTCCTACCCCAATTTCC | TTGGTCCTTAGCCACTCCTTC |
| IL-1b | AATACCACTTGTTGGCTTA | TGTGATGTTCCCATTAGAC |
