# Supplementary material for: Laminaria japonica polysaccharide mitigates acute neuroinflammation in cerebral ischemia-reperfusion injury through Csf3-modulated pathways
Source: Front Immunol. 2026 Apr 23;17:1801746. doi: 10.3389/fimmu.2026.1801746 (PMC13149078; doi:10.3389/fimmu.2026.1801746)
Supplement: Supplementary file 1 [file DataSheet1.zip › Supplementary Files/Table S3.DOCX]

Table S3. Monosaccharide Composition and Molar Ratios of Purified Laminaria japonica Polysaccharide (LJP)

| Monosaccharide Component | Abbreviation | Molar Ratio | Relative Content (%)^1^ |
| --- | --- | --- | --- |
| Fucose | Fuc | 0.506 | 53.3 |
| Galacturonic acid | GalA | 0.204 | 21.5 |
| Glucuronic acid | GlcA | 0.106 | 11.2 |
| Mannose | Man | 0.088 | 9.3 |
| Arabinose | Ara | 0.045 | 4.7 |

¹Relative content was calculated based on the molar ratio of each monosaccharide to the total sum of all monosaccharide molar ratios (total = 0.949), expressed as a percentage.
